# Biochemical and genome sequence analyses of *Megasphaera* sp. strain DISK18 from dental plaque of a healthy individual reveals commensal lifestyle

**DOI:** 10.1038/srep33665

**Published:** 2016-09-21

**Authors:** Nayudu Nallabelli, Prashant P. Patil, Vijay Kumar Pal, Namrata Singh, Ashish Jain, Prabhu B. Patil, Vishakha Grover, Suresh Korpole

**Affiliations:** 1 CSIR-Institute of Microbial Technology, Chandigarh, India; 2 Dr. Harvansh Singh Judge Institute of Dental Sciences and Hospital, Panjab University, Chandigarh, India

## Abstract

Much of the work in periodontal microbiology in recent years has focused on identifying and understanding periodontal pathogens. As the majority of oral microbes have not yet been isolated in pure form, it is essential to understand the phenotypic characteristics of microbes to decipher their role in oral environment. In this study, strain DISK18 was isolated from gingival sulcus and identified as a *Megasphaera* species. Although metagenomics studies revealed *Megasphaera* species as a major group within the oral habitat, they have never been isolated in cultivable form to date. Therefore, we have characterized the DISK18 strain to better understand its role in the periodontal ecosystem. Strain *Megasphaera* sp. DISK18 displayed the ability to adhere and self-aggregate, which are essential requisite features for inhabiting and persisting in oral cavity. It also coaggregated with other pioneer oral colonizers like *Streptococcus* and *Lactobacillus* species but not with *Veillonella*. This behaviour points towards its role in the ecologic succession of a multispecies biofilm as an early colonizer. The absence of virulence determining genes as observed in whole genome sequence analysis coupled with an inability to degrade collagen reveals that *Megasphaera* sp. strain DISK18 is likely not a pathogenic species and emphasizes its commensal lifestyle.

The ‘‘oral microbiome’’ represent an ecological community of mutualistic, symbiotic, commensal and pathogenic microorganisms which are significant determinants of oral health and disease^1^. Many dental diseases like caries or periodontitis are the result of dysbiosis of microbial communities in dental plaque^2^. After initial colonization, commensal bacterial species tend to persist in the mouth for years and co-evolve with host, maintaining the health of the host by preventing access of pathogenic bacteria and stimulating the immune response^3^,^4^. In fact, dental caries are a result of disequilibrium in acid and alkali producing bacteria present in dental plaque/environment^2^. The current paradigm of microbial dysbiosis has highlighted the significance of resident microflora in the maintenance of oral health^2^,^4^.

The use of advanced genomics and molecular biology techniques have revolutionized studies examining the structural and functional complexity of oral microflora. In particular, culture-independent molecular methods using 16S rRNA gene sequence analysis have identified numerous bacterial species in oral environment (http://www.homd.org)^5^. Most of these species belong to six major phyla including *Firmicutes*, *Bacteroidetes*, *Proteobacteria*, *Actinobacteria*, *Spirochaetes*, *Fusobacteria*. Other common strains that have been identified include *Euryarchaeota*, *Chlamydia*, *Chloroflexi*, *Synergistetes* and *Tenericutes*. Interestingly, strains also represented the uncharacterized phyla like SR1 and TM7^1^,^5^,^6^. Even though oral ecosystem found to harbour such vast microbial diversity, till date only about 280 bacterial species have been isolated by cultivable methods, characterized and formally named^1^,^5^. Owing to the stringent growth conditions and technical difficulties, only few microbes have been cultivated from the periodontal environment. *Megasphaera* is one such genus, strains of which have been frequently identified by culture-independent analysis and reported to play an important role in human oral cavity^1^,^5^,^7^,^8^,^9^. Indeed, *Megasphaera* spp. are reported to be present in greater proportions in above mentioned studies on microbial analysis of periodontal disease conditions by metagenomics approaches, raising the possibility of its role as a disease associated microbe (periopathogen). Nevertheless, recently it is found that the *Megasphaera* is overrepresented in diseased conditions^10^. In the present study we have isolated a bacterial strain designated as DISK18 from a subgingival plaque sample of a healthy human oral cavity, which is identified to be a member of the genus *Megasphaera* and subsequently characterized to understand the nature of strain and adaptation ability to oral/periodontal environment to understand its role in the oral environment.

## Results

### Identification of bacterial isolates and characterization of strain DISK18

**Figure 1:**
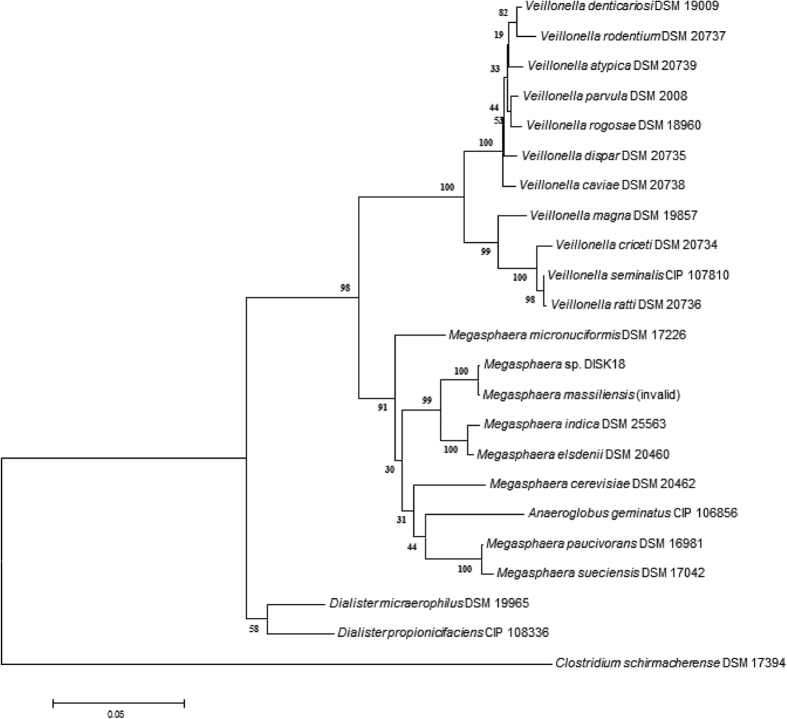
Phylogenetic tree based on 16S rRNA gene sequence using neighbour-joining algorithm. Strain DISK18 formed a distinct cluster with members of the genus *Megasphaera*, and shared a clade with *M. massiliensis* strain NP3^T^, which is an invalid species. A consensus tree was made based on 1000 replications and bootstrap values are expressed as percentage. Bar, 0.05 substitutions per site. *Clostridium schirmacherense* DSM 17394 was used as an outgroup.

Few colonies were isolated upon plating subgingival sample on different media. Based on 16S rRNA gene sequence blast analysis, the genotypic identification of isolates DIGK4C, DISK18, DISK25 and DISK26 revealed significant identity with species of genera *Veillonella*, *Megasphaera*, *Streptococcus* and *Lactobacillus*, respectively. Except *Megasphaera*, members of all other genera have been isolated in cultivable form from oral environment, therefore, *Megasphaera* sp. strain DISK18 was studied in detail by growing it in an anaerobic chamber with a gas phase of N_2_/CO_2_/H_2_ (80:15:5). Strain DISK18 formed opaque colonies with undulate margin and irregular configuration on reinforced clostridial agar medium. Microscopic examination revealed strain DISK18 as non-spore forming and non-motile cocci that stained Gram-negative. It grew well between 30–42 °C temperature with an optimum and maximum growth of 37 and 45 °C, respectively. Growth observed between pH 6.0–8.0 with optimal being 7.0. It grew up to a concentration of 1.5% (w/v) NaCl, however, optimal NaCl concentration for growth was 0.5%. The strain DISK18 showed negative reaction for casein and gelatine hydrolysis. Weak acid production was observed from sucrose but not with dextrose, arabinose, mannose, mannitol, inositol, inulin, fructose, rhamnose, galactose, lactose, maltose or trehalose. It showed a positive reaction for the assimilation of urea. It was also sensitive to the antibiotics kanamycin, erythromycin, tetracycline, rifampicin, chloramphenicol, penicillin, ampicillin, streptomycin and gentamycin but showed resistance to vancomycin. The fatty acid profile of the strain differed with other species of the genus *Megasphaera* as it displayed presence of C_20:1_ ω7c, C_18:3_ ω6c and C_17:1_ is oω5c as major fatty acids. Polar lipid analysis of strain DISK18 revealed amino phospholipids as the major lipids and presence of unknown lipids were also observed (Supplementary Figure S1). Further, phylogenetic analysis of 16S rRNA gene sequence of strain DISK18 showed high identity with *Megasphaera massiliensis* NP3^T^ (99.73%), however, this species was found to be an invalid species of the genus *Megasphaera* and not included in list of prokaryotic standing nomenclature. The next phylogenetic close relative was *M. indica* NMBHI10^T^ with an identity of 96.62%. Similarly, the *rpoB* gene sequence analysis of strain DISK18 also displayed high identity with *M. massiliensis* DSM 26228^T^. Consequently, strain DISK18 formed a coherent cluster with closely related species including *M. massiliensis* NP3^T^*, M. indica* NMBHI10^T^ and *M. elsdenii* LC1^T^ with significant bootstrap value and shared a clade with *M. massiliensis* NP3^T^ in neighbour–joining phylogenetic tree constructed using 16S *rRNA* (Fig. 1). Similar pattern observed in phylogenetic tree constructed with available *rpoB* gene sequences.

### Coaggregation of *Megasphaera* sp. strain DISK18 with other oral isolates

**Figure 2:**
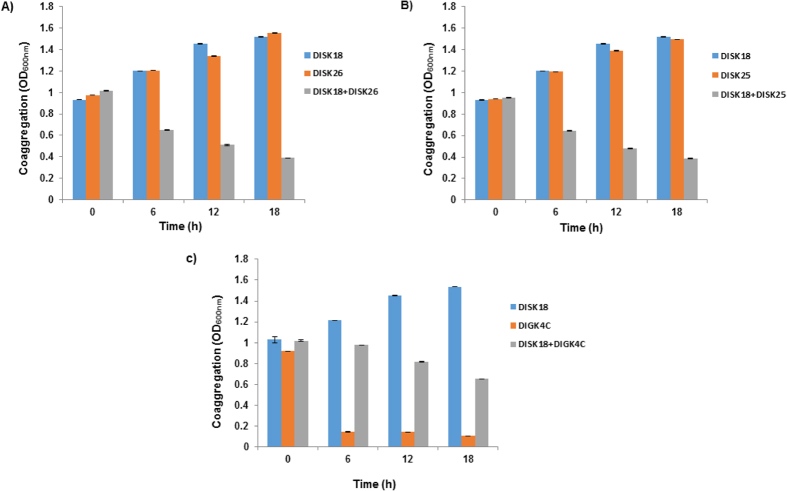
Coaggregation of *Megasphaera* sp. strain DISK 18 with other dental plaque isolates. (**A**) Coaggregation with *Lactobacillus* sp. strain DISK26. (**B**) Coaggregation with *Streptococcus* sp. strain DISK 25. (**C**) Coaggregation with *Veillonella* sp. strain DIGK4C. Error bars indicate the standard deviation (SD) values obtained for triplicate experiments.

The ability of *Megasphaera* sp. strain DISK18 to self-aggregate and coaggregate with other oral isolates including *Streptococcus* sp. strain DISK25 *Lactobacillus* sp. strain DISK26 and *Veillonella* sp. strain DIGK4C was investigated. The results displayed similar self-aggregation ability of *Megasphaera* sp. strain DISK18 compared to *Lactobacillus* sp. DISK26 or *Streptococcus* sp. DISK25 (Fig. 2A,B). However, sharp decrease in OD_600_ observed for *Veillonella* sp. strain DIGK4C (Fig. 2C) reveals highest self-aggregation ability. The coaggregation pattern of efficient strains pairs were examined by interacting the strain DISK18 with each of the other isolate. We found in results that significant coaggregation occurred between the strains *Megasphaera* sp. DISK18 and of *Lactobacillus* sp. DISK26. Similar results were also found with *Streptococcus* sp. DISK25. In contrast to self-aggregation ability of individual strain, the coaggregation efficiency of *Veillonella* sp. DIGK4C with *Megasphaera* sp. strain DISK18 revealed substantial reduction (Fig. 2C).

### Effect of sugar, temperature, pH and proteases on self and co aggregation

**Figure 3:**
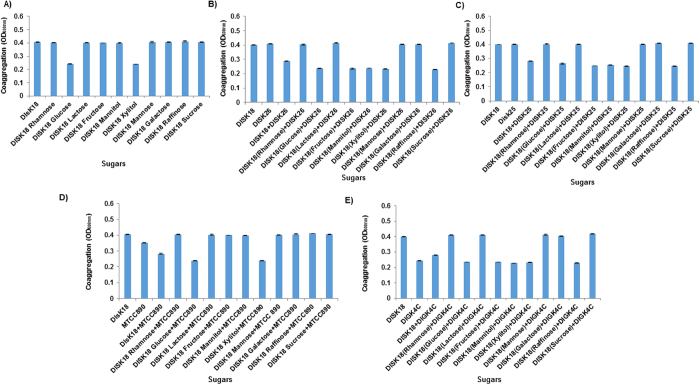
Effect of sugars on self-aggregation and coaggregation of *Megasphaera* sp. strain DISK18. (**A**) Self-aggregation of *Meghasphaera* sp. strain DISK18. (**B**) Coaggregation with *Lactobacillus* sp. strain DISK26. (**C**) Coaggregation with *Streptococcus* sp. strain DISK25. (**D**) Coaggregation with *S. mutans* MTCC 890. (**E**) Coaggregation with *Veillonella* sp. strain DIGK4C. The data shown is representing three individual experiments and the error bar represents standard deviations (SD).

**Figure 4:**
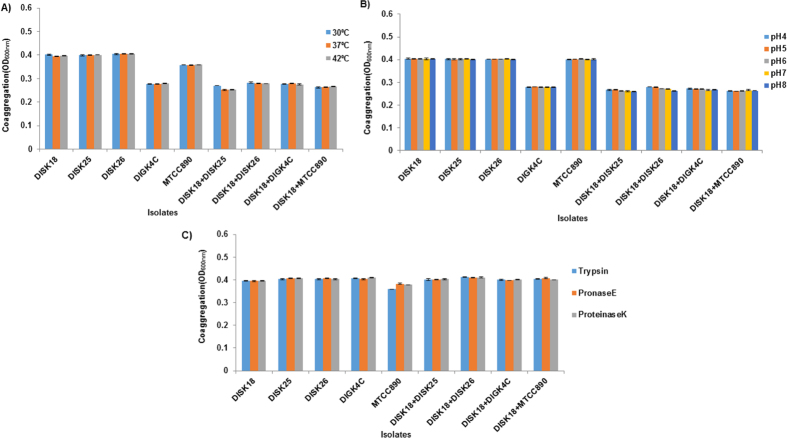
Influence of physiological conditions and proteolytic enzymes on coaggregation of *Megasphaera* sp. strain DISK18 with other oral isolates. (**A**) Effect of temperature on coaggregation. (**B**) Coaggregation under different pH conditions. (**C**) Influence of protease enzymes on coaggregation. Error bars indicate the standard deviation (SD) values obtained for triplicate experiments.

Among the various sugars used to test their influence on self-aggregation or coaggregation, glucose and xylitol were found to increase the self-aggregation ability of *Megasphaera* sp. strain DISK18 (Fig. 3A). The coaggregation between pairs of *Megasphaera* sp. strain DISK18 and different isolates was found to increase in the presence of glucose, fructose, mannitol, xylitol and raffinose (Fig. 3B,C,E). Though similar trends were observed for *Megasphaera* strain DISK18 and *S. mutans* MTCC890, they differed with respect to the presence of galactose and raffinose. While galactose increased the coaggregation between the pair, raffinose did not show any effect (Fig. 3D). Increase in temperature up to 42 °C did not show any influence on self-aggregation of strains but minor reduction observed in coaggregation for different pair combinations (Fig. 4A) and no self-aggregation or coaggregation was observed at 80 °C temperature (data not shown). Self-aggregation or coaggregation at different pH values did not show any differences of any oral isolates or their combinations (Fig. 4B). Additionally, isolates treated with proteolytic enzymes trypsin, pronase E or proteinase K also did not show any significant alterations in either self-aggregation or coaggregation (Fig. 4C).

### Surface hydrophobicity analysis of the isolates

Since property of hydrophobicity in bacteria is associated with biofilm formation^11^, we have tested the surface hydrophobicity of all isolates using chloroform, diethyl ether and hexane. While *Megasphaera* sp. strain DISK18, *Lactobacillus* sp. DISK26 and *Streptococcus* sp. DISK25 isolates revealed higher affinity to chloroform compared to *Veillonella* sp. strain DIGK4C (Supplementary Figure S2). *Megasphaera* sp. strain DISK18 showed lesser affinity to hexane and diethyl ether.

### Biofilm formation ability of *Megasphaera* sp. strain DISK18

**Figure 5:**
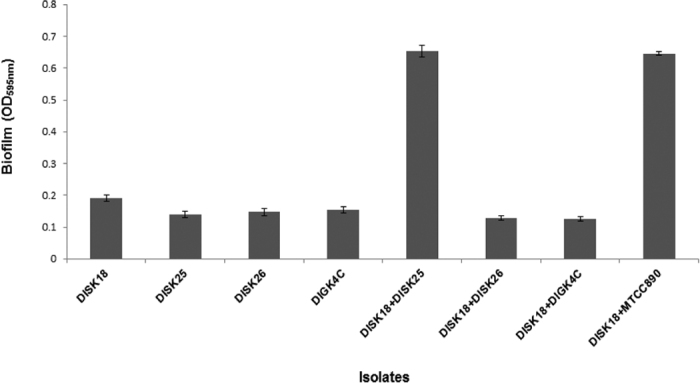
Biofilm formation ability of individual oral isolates and in combination with *Megasphaera* sp. strain DISK18. The biofilm was quantified by crystal violet assay and the values obtained upon subtraction with background control. The data shown is representing three individual experiments and error bar represents standard deviations (SD).

**Figure 6:**
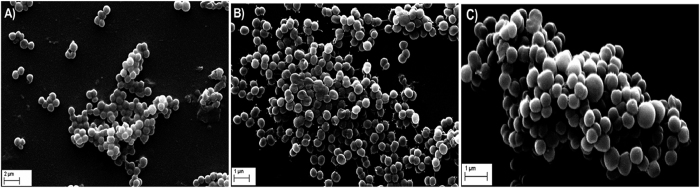
Scanning electron microscopic analysis of biofilm formation by *Megasphaera* sp. strain DISK18. (**A**) Initial stage of biofilm observed after incubation of 3 h. (**B**) Formation of biofilm with pili like appendages after 6 h incubation. (**C**) Matured biofilm upon incubation of 12 h.

Based on the results obtained for self-aggregation, coaggregation and hydrophobicity analysis, biofilm formation was assayed for the *Megasphaera* sp. strain DISK18 alone as well as in mixed cultures with other oral isolates to test the multispecies biofilm formation efficiency. Results of the crystal violet microtiter plate biofilm formation assay revealed *Megasphaera* sp. strain DISK18 could form biofilm with moderate-density as a single culture and formed efficient biofilm in combination with *Streptococcus* sp. strain DISK25 and MTCC 890 (Fig. 5). Formation of biofilm by *Megasphaera* sp. strain DISK18 was also confirmed with electron microscopy experiment where the formation of pilus like appendages clearly observed during initial phase of biofilm and with the increase in incubation duration they extended towards each other bacterial cells (Fig. 6A,B). The mature biofilm contained multiple layers of cells attached with each other (Fig. 6C).

### Volatile sulfur compounds (VSC) production by *Megasphaera* sp. strain DISK18

To analyse the role of *Megasphaera* sp. strain DISK18 in halitosis, we tested the production of volatile sulfur compounds (VSC). Though colonies were formed within 24 h on differential agar, they stained black only after 72–96 h incubation. Accordingly, delayed VSC production was observed by microscopic assay (Supplementary Figure S3) and the same was confirmed with differential agar method. However, quantification of VSC levels was not performed.

### Fluoride resistance of *Megasphaera* sp. strain DISK18

We have tested the tolerance ability of *Megasphaera* sp. strain DISK18 towards different fluoride concentrations as fluoride is one of the most common ion available in local environment from various oral hygiene products used in oral cavity.Results showed that it could tolerate up to 1000 μg/ml concentration of fluoride and no growth was observed above this concentration.

### Genome features of *Megasphaera* sp. strain DISK18

**Figure 7:**
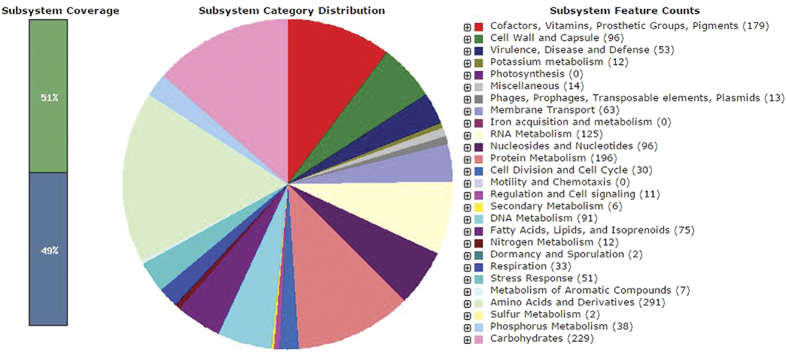
Subsystem category distribution of major protein coding genes of *Megasphaera* sp. strain DISK18 as annotated by Rapid Annotation System Technology (RAST) server^12^. Bar chart (at leftmost) shows the subsystem coverage in percentage (green bar corresponds to percentage of proteins included). The pie chart depicting percentage distribution of 20 most abundant subsystem categories in strain DISK18. Representative slices in pie chart indicated with colour in right panel along with count of subsystem feature.

To understand the genetic structure of *Megasphaera* sp. strain DISK18 towards the observed phenotypic characters, we sequenced the whole genome of the isolate DISK18 and reads were de novo assembled by using CLC Genomics Workbench 7.5 (CLC Bio-Qiagen, Aarhus, Denmark) and further annotated by using Rapid Annotations using Subsystems Technology (RAST) server^12^ as mentioned in methods. The genome size of the *Megasphaera* sp. DISK18 was 2.4 Mbp with 50.06% of G+C content which assembled into 65 contigs. General genome features and assembly statistics of the strain DISK18 are provided in Table 1. Genome annotation based on RAST analysis predicted there were 2297 genes amongst which 2235 were coding sequences (CDS). The subsystem distribution, representing a collection of functionally related protein families and distribution statistics of CDS in the genome of *Megasphaera* sp. DISK18 revealed a total of 324 subsystems. Amongst these, amino acid and derivatives subsystem featured with largest number (291) of the assigned CDS. Other major subsystems annotated were carbohydrate (229), protein metabolism (196), cofactors, vitamins, prosthetic groups and pigments (179) and RNA metabolism (125). While only two CDS represented the sulfur metabolism, no CDS involved in motility or chemotaxis were annotated (Fig. 7). There were also eight clustered regularly interspaced short palindromic repeats (CRISPR) observed but no secondary metabolite gene clusters were predicted in the genome of the *Megasphaera* sp. strain DISK18.

### Genome based comparison with other *Megasphaera* species

**Figure 8:**
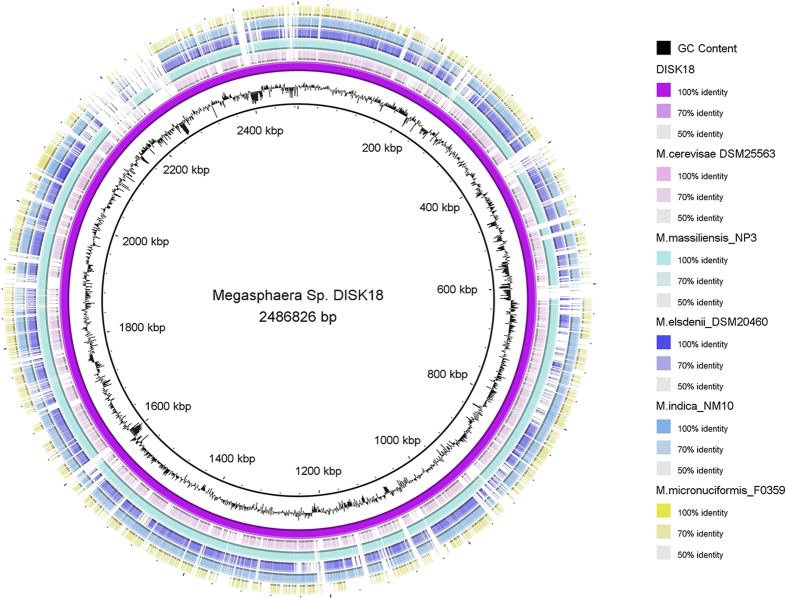
The whole genome based comparison of the *Megasphaera* sp. strain DISK18 with closely related members of the genus *Megasphaera* using BLAST Ring Image Generator^16^. *Megasphaera* sp. strain DISK18 is used as reference genome. Each ring represents a single genome. From the inside out, the inner ring represents GC content followed by *Megasphaera* sp. strain DISK18. From third circle to next five rings represent the genomes of *M. cerevisae* DSM 25583, *M. massiliensis* NP3, *M. elsdenii* DSM 20460, *M. indica* NM10 and *M. micronuciformis* F0350, respectively.

The genome sequence of *Megasphaera* sp. strain DISK18 was compared with other *Megasphaera* species whose genome sequences are available at NCBI database. For assessment of genome similarity we used Average Nucleotide Identity (ANI) and digital DNA –DNA hybridization (dDDH). The whole genome ANI value between *Megasphaera* sp. strain DISK18 and close relatives including *M. massiliensis* DSM 26228^T^ 13, *M. indica* DSM 25563^T^ 14 and *M. elsdnii* DSM 205460^T^ 15 displayed 96.5%, 80.5% and 80.3%, respectively. The digital DNA-DNA hybridization (dDDH) of strain DISK18 with above mentioned strains revealed an identity of 71.7%, 24.4% and 24.1%, respectively. The ANI and dDDH values for strain *Megasphaera* sp. DISK18 with *M. cerevisiae* DSM 25563^T^ were found to be much lower than above mentioned strains. Further, whole genome comparison of all above mentioned *Megasphaera* strains was performed using BLAST Ring Image Generator (BRIG) tool^16^ (Fig. 8) using genome of strain DISK18 as a reference. The comparison revealed a large scale variation in the genomes of different strains with *Megasphaera* sp. strain DISK18 that were further compared using RAST annotation.

### Gene content analysis of *Megasphaera* sp. strain DISK18 whole-genome sequence

Detailed analysis of the subsystems features revealed that the strain DISK18 contained numerous genes involved in combating oxidative stress. For example, genes encoding glutathione peroxidase, lactoylglutathione lyase, hydroxyacyl glutathione hydrolase, rubreythrin and superoxidase reductase were present. In addition, rex and PerR systems that are involved in anaerobic metabolism were also found on genome^17^. Several annotated genes of *Megasphaera* sp. strain DISK18 genome sequence were involved in the biosynthesis of Vitamin B complex, cofactors and prosthetic groups. Interestingly, *Megasphaera* sp. strain DISK18 did not contain virulence factors like *tpr*, *prtC* (collagenase), *rgpA* (arg-gingipain), *fimA* and *mgl1* or any other pathogenic genes involved in development of oral diseases as observed for strains of the genus *Porphyromonas*, particularly, dental pathogen *P. gingivalis*^18^. However, strain DISK18 contained antibiotic resistance genes including beta lactam, fluroquinolone and vancomycin resistance genes. Additionally, efflux pump like cmeABC and mexC-mecD efflux pump, Acriflavine resistance (*acrA* and *acrB* genes) belonging to RND family and YdhE/NorM efflux pump of MATE family that are known to impart antibiotic, heavy metal and bile salts resistance^19^ were also observed in genome of *Megasphaera* sp. strain DISK18. Remarkably, many genes encoding adhesins such as auto transporter adhesions, *AidA* adhesion and fibronectin/fibrinogen-binding protein that are also involved in biofilm formation^20^ were found in the annotated genome of strain DISK18. Moreover, type II/IV secretion system RcpA/CpaC associated with Flp pilus assembly and involved in biofilm formation was also present^21^. Genes regulating the peptidoglycan synthesis and exopolysaccharide production, primarily mannose and rhamnose containing exopolysaccharide glycans were found in the genome sequence annotation. Pathway analysis using KEGG showed the presence of fewer/partial metabolic genes involved in sulfur reduction process indicating the ability of *Megasphaera* sp. strain DISK18 to contribute in sulfur reduction process. However, these genes included only sulfate adenylyl transferase subunit 1 and subunit 2, adenylyl sulfate reductase beta-subunit and adenylyl sulfate reductase alpha-subunit. Other related genes present were serine acetyltransferase, ferredoxin–sulfite reductase, cysteine synthase, homoserine O-succinyltransferase, O-acetylhomoserine sulfhydrylase. Unusually, *Megashpera* sp. strain DISK18 contained gene *crcB* associated with the recently discovered fluoride riboswitch, which contributes towards the fluoride resistance.

## Discussion

The microflora of the oral cavity reside in different habitat niches with in the oral cavity according to the local environmental conditions^3^. While most of the microbes in the mouth are beneficial, selected few are harmful and cause damage to teeth^4^. The narrow space between the tooth surface and adjacent gingiva (i.e. the subgingival area) is regarded primarily as an anaerobic zone, harbouring subgingival dental plaque, usually containing periodontal pathogenic microorganisms. A recent culture independent study using a species-specific PCR primer set for the detection of *Megasphaera* suggested that members of *M. micronuciformis*, which have been isolated from liver abscess and pus samples^22^, are widely distributed in the oral cavities of the Japanese population^9^. Other studies based upon culture-independent 16S rRNA gene cloning and sequence analysis of biofilms associated with chronic periodontitis have reported elevated levels of the genus *Megasphaera* including the oral clones like BB166, MCE3_141 and BS073^7^,^8^. These were reported to be essentially involved in periodontitis disease as *Megasphaera* clone BB166 was frequently observed in chronic periodontitis patients^7^,^8^. In spite of these observations, there is no direct evidence demonstrating the role of *Megasphaera* species in disease. The genus Megasphaera includes 6 validly published species (*M. cerevisiae, M. elsdenii, M. indica, M. micronuciformis, M. paucivorans* and *M. sueciensis*) isolated from various sources such as the rumen, spoiled beer and human clinical specimens^14^,^15^,^22^,^23^,^24^. Although *Megasphaera* like phylotypes have been found in abundant quantities during metagenomic analysis of oral samples^7^,^8^,^9^, but there has been no strain isolated in culture or characterized directly from oral environment. Thus, in this study we have performed a series of experiments including the whole genome sequence analysis to decipher the role of an isolated *Megasphaera* sp. strain DISK18, in dental plaque oral ecology. Though strain DISK18 showed high sequence identity with *M. massiliensis* DSM 26228, the latter was isolated from different environment (fecal sample of an HIV infected patient) and is not a validly published strain. Therefore, *Megasphaera* sp. strain DISK18 might be a potential novel species of the genus *Megasphaera* based on its differences with the closest validly published species *M. indica* DSM 25563.

The inability to cause a disease by *Megasphaera* sp. strain DISK18 was established by comparing the whole genome sequence against the genome of a known periopathogen, *P. gingivalis*. As a result, whole genome sequence of strain DISK18 did not show any homologs to major virulence genes including *tpr*, *prtC (collagenase)*, *rgpA (arg-gingipain)*, *fimA* or *mgl1* that are associated with pathogenesis in *P. gingivalis*^18^,^25^. The 53 virulence related genes identified during annotation were found to be antibiotic resistance genes involved in drug resistance, nevertheless, *Megasphaera* sp. strain DISK18 was found to be sensitive to most of the antibiotics except vancomycin. The degradation of dento-epithelial collagen tissue by collagenase activity leads to the formation of a periodontal pocket with low redox levels, thus favouring the growth of anaerobic species and leads to periodontal disease resulting in loss of alveolar bone^26^. However, the inability of strain DISK18 to hydrolyse gelatin or collagen and the absence of any genes encoding collagenase or gelatinase enzymes clearly suggests that *Megasphaera* sp. strain DISK18 is not involved in periodontal disease. Interestingly, the close relatives like *M. massiliensis* and *M. elsdenii* have demonstrated the ability to hydrolyse gelatin^13^.

A series of experiments was carried out to check the adaptability of the isolated strain in the oral/periodontal environment. Since adherence to naturally available surfaces in the oral cavity is a prime requisite for any microbe inhabiting oral cavity^27^, the ability of the strain DISK18 to adhere was investigated through cell surface hydrophobicity experiments along with other oral isolates. It is pertinent to mention that all of the isolates showed significant affinity to nonpolar solvents indicating their hydrophobic nature. In biological systems, hydrophobic interactions are usually considered to be the strongest of all long-range non-covalent interactions and play an important role in the adhesion process^11^. The bacteria showing hydrophobic surfaces have been demonstrated to be more adherent to polystyrene coated surfaces, than hydrophilic cells *in vitro*^28^.

The majority of oral microbial flora forms biofilms of complex communities in dental plaque that leads to successful colonization^29^. This multispecies biofilm often displays characteristics like adherence and coaggregation with bacterial strains within the biofilm. In fact, this is considered as one of the central mechanisms of plaque formation^30^,^31^. Dental plaque is a result of coaggregation, a process where genetically distinct bacteria become attached to one another, thereby developing a complex multispecies biofilm^30^. Coaggregation between pairs of bacteria was previously shown as a highly specific reaction^31^ and often observed as a common phenomenon between strains of diverse genera found within dental plaque^32^,^33^. In this study *Megasphaera* sp. strain DISK18 coaggregated well with strains of *Streptococcus* and *Lactobacillus* which are considered as early colonizers of the tooth surface during plaque formation. This observation suggests the potential role of isolate DISK18 in forming multispecies biofilm with these colonizing strains in oral cavity. In contrast, despite displaying self-aggregation ability, strains *Megasphaera* sp. DISK18 and *Veillonella* sp. DIGK4C did not show coaggregation. This suggests that *Megasphaera* and *Veillonella* species may not coexist together, but may play diverse roles in dental plaque ecology via interactions with other oral inhabitants.

Knowledge of coaggregation profiles is helpful in understanding the vital role of coaggregation in bacterial succession and colonization of hard tissue surfaces. To analyse the nature of coaggregation, a variety of experiments were carried out under different temperatures, sugars and treatment with protease. The coaggregation was influenced by different sugars, further it was inhibited by the addition of lactose, galactose and sucrose. The inhibition by sugar might be mediated by lectin and carbohydrate interactions, since inhibition was driven by specific sugars, as a result of the presence of receptor polysaccharides and reciprocal adhesins^31^. The coaggregation ability of *Megasphaera* sp. DISK18 with *Lactobacillus* and *Streptococcus* isolates may plausibly be related to the utilization of lactic acid, a metabolic end product from these species, as a nutrient. This may contribute to a symbiotic relationship between these two species and *Megasphaera* sp. strain DISK18 creating a simple food chain or web within the interacting species. Similar interactions have been documented for *Veillonella* species with *Streptococcus*^34^, therefore, there may be a competitive interaction that exists between the two strains that do not co-aggregate. Interestingly, the inability to coaggregate between *Megasphaera* sp. DISK18 and *Veillonella* sp. DIGK4C could be attributed to competition for the similar binding sites or same coaggregation partner due to homology on their surface receptors (as both strains belong to the class *Veillonellaceae*) that resulted in lowering the coaggregation in comparison to self-aggregation^31^,^34^.

*Megasphaera* sp. DISK18 showed moderate biofilm formation ability individually, but strong mixed-species biofilm formation was observed when combined with *Streptococcus* sp. DISK25 isolate (Fig. 5). It also displayed an ability to form biofilm with other strains of *Streptococcus*. In line with this, we found that, the genome sequence also contained several genes that are involved in biofilm formation such as fimbriae, autotransporters and AIDA genes. These genes are known for adhesion of Gram negative anaerobic bacteria^20^. Among these genes, AIDA is an autotransporter glycoprotein, a potential bacterial adhesion found in *E. coli*^35^ that mediates bacterial aggregation via intercellular self-recognition. Essentially, it is reported to mediate bacterial attachment to a variety of animal and human cell lines^36^. A heptosyltransferase known as autotransporter adhesin heptosyltransferase (AAH) involved in modification of the AIDA adhesion or binding to mammalian cells is also found in the genome sequence of *Megasphaera* sp. strain DISK18. Further, SEM analysis of biofilm revealed the cellular interactions via fibril like threads between the cells (Fig. 6B), which possibly are kind of pilli associated with the cell surface appendages in isolate *Megasphaera* sp. DISK18.

The microflora of oral cavity experiences constant changes in pH, oxygen tension, and nutrient availability, in response to variations in diet and a variety of physical and nutritional interactions within the biofilm community. The complexity of the periodontal microflora combined with a host of variable environmental parameters make this system particularly challenging for bacteria to adapt and flourish as a commensal or resident flora. Gingival sulcus provides a relatively anaerobic environment, so the bacteria residing in this region are subjected to varying concentrations of oxygen depending upon the composition and age of the biofilm. The MoxR family of AAA+ ATPases, widespread among bacteria and archaea are present in *Megasphaera* sp. strain DISK18. MoxR proteins have been found to be important modulators of multiple stress response pathways in different organisms. Many stress response proteins like chaperone dnaK gene cluster, heat shock protein 33, Grp E, Hsp 60 GroEL co-chaperone GroES were also identified on genome sequence of *Megasphaera* sp. strain DISK18. Phosphate starvation protein Pho H and stringent starvation a & b related to carbon starvation were also present. Associated with the gene clusters of particular significance are the genes ATP phosphoribosyltransferase (EC 2.4.2.17) which may have a role in histidine biosynthesis in *Megasphaera*. Similar mechanisms have been documented in *Lactobacillus casei*, in response to acid adaptation^37^. It was suggested that the histidine operon resulted in increased intra-cellular levels of histidine which may be contributing to intracellular buffering capacity as the pKa value of the imidazole groups of histidine and histidine-containing peptides is near 6.0^38^. These stress response genes may help the organism by the induction of many proteins in response to a variety of environmental stress conditions and have significant impact on bacterial physiology and survival.

The fluoride riboswitch, an adaptive strategy of oral microbes to deal with fluoride ion^39^, which is present in majority of oral hygiene products. Though members of the family *Veillonellaceae* were unaffected by fluoride presence^40^, they were never reported to contain fluoride riboswitch. This is the first report that reveals adaptive strategies developed in *Megasphaera* strain DISK18 compared to its closest phylogenetic relatives. Moreover, in *Megasphaera* sp. strain DISK18 genome we also observed the gene crcB located upstream of the sodium solute transporter and type IV secretion system. The presence of genes encoding sulfate reductase enzymes involved in sulfur reduction confirmed the production of VSC, though, in low quantities. Similarly, the presence of enzyme carbamate kinase, which mediates the terminal step in ammonia degradation in the strain *Megasphaera* sp. DISK18 suggests its ability to neutralize the periodontal environment and reduce the damage caused by cariogenic bacteria under acidic conditions. Many oral bacteria like *S. mitis*, *S. pneumoniae*, *S. gordonii*, and *S. cristatus* express arginine deiminase^41^,^42^, which is the first enzyme in the arginine deiminase system that converts arginine to ammonia, ornithine, and carbon dioxide. Similarly, strain DISK18 also produced ammonia with the help of enzyme arginine deaminase.

### Clinical relevance of *Megasphaera* as a commensal organism to the host

Most of the normal resident bacteria of the oral environment benefit the host through the production or utilization of metabolites produced through various metabolic pathways. These range from fermentation end products (mostly short chain fatty acids), vitamins and co-factors to numerous other bio-molecules^14^. In the present study, we identified many genes responsible for such potentially beneficial effects in the genome of *Megasphaera* sp. strain DISK18 which could contribute to the production of important metabolites, vitamins and cofactors. Usually health associated bacteria do not survive in very low pH^41^ which is in line for *Megasphaera* sp. strain DISK18 as well, which resides in an alkaline pH, so, it is quite plausible that *Megasphaera* spp. may have a beneficial role to the host health via its lactate utilization and alkali generation activities, and compete with the growth of pathogenic organisms.

## Conclusion

Though newly developed tools of metagenomics have allowed us to understand the complexity of the oral microbiome, the traditional tools to isolate and culture individual bacterial community members are essentially required. Cultivation and culturing methods help to extend our understanding of the ecologic and metabolic characteristics or behaviours of newly identified microorganisms and to decipher their role in oral health and disease. Thus, present study reports the characterization of an oral isolate *Megasphaera* sp. DISK18 along with whole genome sequence emphasizing its potential role as a commensal in the oral cavity. In particular, the range of optimal temperature, pH, oxidative stress, antibiotics and fluoride resistance, metabolic end products coupled with absence of pathogenic nature supported by the whole genome sequence data highlight its commensal life style.

## Material and Methods

### Collection of sample

The subgingival plaque sample was collected from a 24 year old female with a healthy oral cavity at Dr. H.S. Judge Institute of Dental Sciences and Hospital, Panjab University, Chandigarh. Study subject was selected based on absence of any specific dental pathology or associated conditions like dental caries, missing tooth or any prosthesis in her mouth. The clinical indicators of periodontal status were recorded and no sites of pocket depth or attachment level measurements >3 mm, absence of overt gingival redness and/ or bleeding on probing were observed ascertaining a clinically healthy status of oral cavity. The purpose of investigation was explained and a voluntary informed consent was obtained from the subject. All procedures involving sample collection from human subjects were approved by Institutional review committee (Dr. H.S. Judge Institute of Dental Sciences and Hospital) and were carried out in accordance with the approved guidelines. The subgingival plaque sample was collected with sterile paper points. Paper points were inserted into gingival sulcus at 6 sites minimally in each quadrant and kept undisturbed for at least 20 seconds. The pooled plaque was subsequently transferred to a serum vial containing reduced transport fluid media. Airtight sealed vials were transported to the laboratory for isolation of bacteria.

### Bacterial strains

All strains, except *S. mutans* MTCC 890, used in the present study were isolated from the gingival plaque samples. Serially diluted samples were plated on different media and incubated under anaerobic atmosphere (80% N_2_, 15%, CO_2_, 5% H_2_). Bacterial strains designated as DIGK4C and DISK18 were isolated from reinforced clostridial agar (Difco), DISK25 from brain heart infusion agar (Difco) and DISK26 from MRS agar (Difco) plates upon incubation at 37 °C for 48. The individual colonies were streaked onto respective media of isolation, purified and preserved as −70 °C glycerol stocks for further studies. Strain *S. mutans* MTCC 890 was obtained from Microbial Culture Collection and Gene bank, CSIR-Institute of Microbial Technology, Chandigarh.

### Genotypic identification of isolates

All strains were identified based on 16S rRNA gene sequence analysis as described earlier^43^. The 16S rRNA gene sequence (~1400 bp) was used for BLAST analysis on EzTaxon database^44^ and closely related sequences were retrieved for manual editing of sequences.

### Phenotypic characterization of strain DISK18

To determine the cell morphology, strain DISK18 was grown on reinforced clostridial agar medium at 30 °C under anaerobic condition. An active culture was assigned for Gram's staining by using Gram-staining kit (Himedia) according to the manufacturer’s instructions. Ability to grow at different temperatures was performed by incubating strain DISK18 at 4, 10, 15, 25, 30, 37, 45 and 50 °C. The growth at different pH range was tested at 30 °C temperature using reinforced clostridial agar as basal medium and adjusting the pH between 4.0–11.0 using different buffered solutions as described earlier^45^. Reinforced clostridial agar medium was supplemented with different concentrations of NaCl (0.5–2.5% w/v with 0.5% interval) to determine salt tolerance of the strain DISK18. Assimilation of different substrates and biochemical characteristics were performed using AN microplates (BIOLOG) and ANC microplates (Vitek2; BioMerieux) following the manufacturer's instructions. Catalase and oxidase activities were determined as described in previous literature^46^. Hydrolysis of esculin, indole, Voges- Proskauer and methyl red test, H_2_S production and nitrate reduction were performed as described by Lanyi^47^. Hydrolysis of casein, gelatin, starch and tween was determined as described by Smibert and Kraig^48^. Further, acid production from different sugars was checked as mentioned earlier^45^. Antibiotic susceptibility of the strain was determined using antibiotic discs supplied by Himedia (India). The total polar lipids were extracted using the protocol described by Minnikin^49^. To determine the membrane lipid content, polar lipids were extracted and separated by two dimensional TLC and sprayed with molybdatophosphoric acid, molybdenum blue and ninhydrin spray reagents to detect total lipids, phospholipids and aminolipids, respectively. Strain DISK18 is deposited at Microbial Type Culture Collection under the accession number MTCC12521.

### Phylogenetic analysis of strain DISK18

For phylogenetic analysis, the 16S rRNA gene sequence of strain DISK18 (1481 bp) was used for BLAST analysis on EzTaxon database. Closely related sequences were retrieved and aligned using ClustalW program^50^. Phylogenetic trees were constructed using the neighbour-joining (NJ)^51^ and maximum likelihood (ML)^52^ methods available in MEGA6 software^53^. Kimura two-parameter^54^ model was used to calculate the evolutionary distance of NJ and ML tree. Search option NNI (Nearest-Neighbour-Interchange) with very strong branch swap filter was used for ML phylogenetic analysis. A discrete gamma distribution was used to model evolutionary rate differences among sites (5 categories (+G, parameter = 0.3358). All positions containing gaps, missing data were eliminated and bootstrap analyses were performed using 1000 replications for both NJ and ML analyses. The 16S rRNA gene sequence is available at NCBI with accession no. LN998020.

### Measurement of surface hydrophobicity

The surface hydrophobicity of all isolates (DISK18, DISK26, DIGK4C and DISK25) was tested using chloroform, diethyl ether and hexane partition method^55^. In brief, approximately 10^9^ cells were washed twice with 3 mM NaCl containing 0.5 mM CaCl2 and suspended in the same solution. An equal volume of cell suspension was mixed with chloroform, hexane or diethyl ether (5 ml each) into a round bottom test tube. The tubes were mixed by vortex mixing and the hydrophobicity was expressed as the solvent phase adsorption percentage that was calculated based on the measurement of the OD_600_ of the cell suspension before and after the assay using formula Hydrophobicity (%) = [1 − (OD600 after vortex mixing/OD600 before vortex mixing)] × 100.

### Sulfate reduction and volatile sulfur compounds (VSC) production

The production of volatile sulfur compounds by *Megasphaera* sp. strain DISK18 was performed by microscopic sulfide assay^56^. Overnight cultures of *Megasphaera* sp. strain DISK18 (0.5 ml) was added to an equal volume of PBS containing sodium thiosulfate (50 mg) and ferrous sulfate (20 mg) and incubated overnight at 37 °C. The culture was observed under the microscope for VSC production. Further, VSC production was also confirmed by differential agar method^57^. Strain DISK18 (50 μl)was mixed with 50 μl of PBS and plated on to a differential agar containing sodium thiosulfate (0.5 g) and ferrous sulfate (0.2 g). Plates were incubated at 37 °C under anaerobic conditions and observed for formation of black colonies as a result of VSC production.

### Bacterial coaggregation assay between bacterial isolates

Isolates including DISK18, DISK26, DIGK4C, DISK25 and *S. mutans* MTCC 890 were grown to late exponential or stationary phase (48–72 h). The cells were harvested by centrifugation of liquid culture at 8000 rpm for 10 min. The pellet obtained was washed twice with coaggregation buffer^32^ (0.1 mM CaCl_2_, 0.1 mM MgCl_2_, 0.15 M NaCl, and 3.1 mM NaN_3_, dissolved in 1 mM Tris adjusted to pH 8) and suspended in the same buffer. The OD_600_ of the each bacterial cell suspension was measured and adjusted equally before performing self-aggregation and coaggregation assays. Equal amounts (2 ml) of each bacterial culture was mixed with strain DISK18 by vortex mixing to test their coaggregating efficiency. Pure bacterial culture of each strain was also incubated under identical condition to test the self-aggregation. Aliquots were obtained at different time intervals and the coaggregation index was calculated^58^. Assays were performed in three independent experiments in triplicates.

### Effect of sugar, protease pH and temperature on coaggregation

The influence of various external factors such as heat treatment, pH and addition of different sugars on the self-aggregation of strain DISK18 and coaggregation in pairs with other oral isolates was tested. For heat treatment, cell pellets obtained from stationary phase grown cultures of all isolates were resuspended in coaggregation buffer and exposed to various temperatures to test self-aggregation or coaggregation. The bacterial cell suspensions incubated at identical temperatures were used to test the effect of pH on coaggregation as described above. Similarly, cells were resuspended in different buffers ranging pH from 4.0 to 8.0 in 1.0 pH interval to test the effect of pH and untreated strains were used as control. To evaluate the effects of different sugars on self-aggregation of strain DISK18 and coaggregation with other oral isolates, cell pellet obtained from culture grown to stationary phase were mixed with 50 mM solutions of fructose, galactose, glucose, lactose, mannitol, mannose, rhamnose, sucrose and xylitol. The effect of proteolytic enzymes was tested by incubating the cell pellet of isolates with individual enzymes like pronase E, proteinase K and trypsin in recommended buffers with a final protease concentration of 2.5 mg/ml. Upon incubation for one hour at 37 °C, cells were washed with coaggregation buffer and resuspended in same buffer. Equal amounts of strain DISK18 was mixed with each of the other isolates and coaggregation was measured as OD_600_ using spectrophotometer.

### Biofilm formation ability

To understand the ability of strain DISK18 to form biofilm, experiments were performed on strain DISK18 alone and also in combination with other isolates to estimate the multispecies biofilm formation efficiency. The isolates were grown anaerobically in reinforced clostridial broth or trypticase soy broth overnight. After incubation the cultures were diluted 1:100 and 100 μl of this cell suspension was transferred to a 96 well-microtitre plate and incubated at 37 °C for 24 h under anaerobic conditions. Reinforced clostridial broth and trypticase soy broth were used as control. Subsequently, the medium was discarded and wells were washed twice with 200 μl of PBS to remove planktonic or loosely attached bacteria and air dried for 30 min. The bound cells were stained with 120 μl of 0.1% crystal violet solution and plates were incubated at room temperature for 15 min. Excess dye was removed by washing the wells with double distilled water vigorously and allowed to dry. The quantification of biofilm formation was performed by adding 125 μl of 30% acetic acid to each well as a solubilizing agent for crystal violet and incubated for 15 min. The quantification of biofilm was done by measuring OD at 595 nm using 30% acetic acid as blank. All biofilm assay experiments were performed in triplicate. Biofilm formation was also performed in 12 well plate and selected biofilms were processed for scanning electron microscopy (SEM) study.

### Whole genome sequence, assembly and annotation

The genomic DNA was extracted and purified using Sambrook method^59^ and also using ZR Fungal*/*Bacterial DNA MiniPrep Kit (Zymo Research, USA) as per manufacturer’s instructions. The DNA was quantified by using *Qubit* 2.0 Fluorometer (Life Technologies) and used for whole genome sequencing. Illumina sequencing libraries were constructed by using the Nextera XT sample preparation kit (Illumina, Inc., San Diego, CA, USA) with dual indexing adaptors from Illumina as per the manufacturer’s guidelines. Sequencing libraries were sequenced by using 2 × 150 bp paired configuration on the in-house Illumina Miseq (Illumina, Inc., San Diego, CA, USA) platform. Illumina reads were de novo assembled using CLC Genomics Workbench 7.5 (CLC Bio-Qiagen, Aarhus, Denmark). The genome was annotated by using NCBI-Prokaryotic genome annotation pipeline and RAST server (htpp://rast.nmpdr.org)^12^. Subsystem functional categorization of the predicted ORFs and visualization was done by using SEED viewer^60^. Average nucleotide identity (ANI) values were determined using the tool Jspecies^61^ and digital DDH was calculated using web tool GGDC 2.0 (http://ggdc.dsmz.de/distcalc2.php). CRISPR repeats were identified using the CRISPR recognition tool^62^. BLAST Ring Image Generator (BRIG) software was used to compare the genome sequences and visualization^16^. The whole genome shotgun project has been deposited at DDDBJ/EMBL/GenBank under accession number LUGJ00000000.

## Additional Information

**Accession codes:** EMBL/DDJB/GenBank accession number for 16S rRNA gene sequence of strain is LN998020 and the genome sequence is LUGJ00000000.

**How to cite this article**: Nallabelli, N. *et al*. Biochemical and genome sequence analyses of *Megasphaera* sp. strain DISK18 from dental plaque of a healthy individual reveals commensal lifestyle. *Sci. Rep.* **6**, 33665; doi: 10.1038/srep33665 (2016).

## Supplementary Material

Supplementary Information

## Figures and Tables

**Table 1:** Comparison of genome features of *Megasphaera* sp. strain DISK18 with close relatives.

Attribute	Strain DISK18	*M. misseliensis* strain NP3	*M. indica* strain DSM25563
Genome Size (bp)	2,486,826	2,661,757	2,615,280
G+C content (%)	50.06	50.20	54.30
Fold Coverage (X)	179	19	74
N50	77144	NA	NA
Contig count	65	114	NA
No. of coding sequences	2,235	2,577	2,432
No. of rRNA operons	5	2	8
No. of tRNA operons	56	61	58
CRISPR repeats	8	7	4
NCBI-GeneBank database accession number	LUGJ00000000	CAVO00000000	APHY00000000
