# Supplementary material for: Biochemical and genome sequence analyses of Megasphaera sp. strain DISK18 from dental plaque of a healthy individual reveals commensal lifestyle
Source: Sci Rep. 2016 Sep 21;6:33665. doi: 10.1038/srep33665 (PMC5030485; doi:10.1038/srep33665)

Biochemical characterization and genome sequence analysis of Megasphaera sp. strain DISK18 from healthy individual dental plaque revealed its commensal lifestyle

Nayudu Nallabelli [1]*, Prashant Patil[1]*, Vijay Kumar Pal[1], Namrata Singh[1], Ashish Jain[2], Prabhu B. Patil[1], Vishakha Grover[2†], Suresh Korpole[1†]

1. CSIR-Institute of Microbial Technology, Chandigarh, India. 2. Dr. Harvansh Singh Judge Institute of Dental Sciences and Hospital, Punjab University, Chandigarh, India.

Total number of pages: 03

Total number of supplementary tables: 00

Total number of supplementary figures: 03

Supplementary Figure S1: Membrane polar lipid analysis of *Megasphaera* sp. strain DISK-18. (A) Total polar lipids stained using molybdatophophoric acid. (B) Detection of amino lipids using ninhydrin reagent. (C) Phospholipids detection using the reagent molybdenum blue.

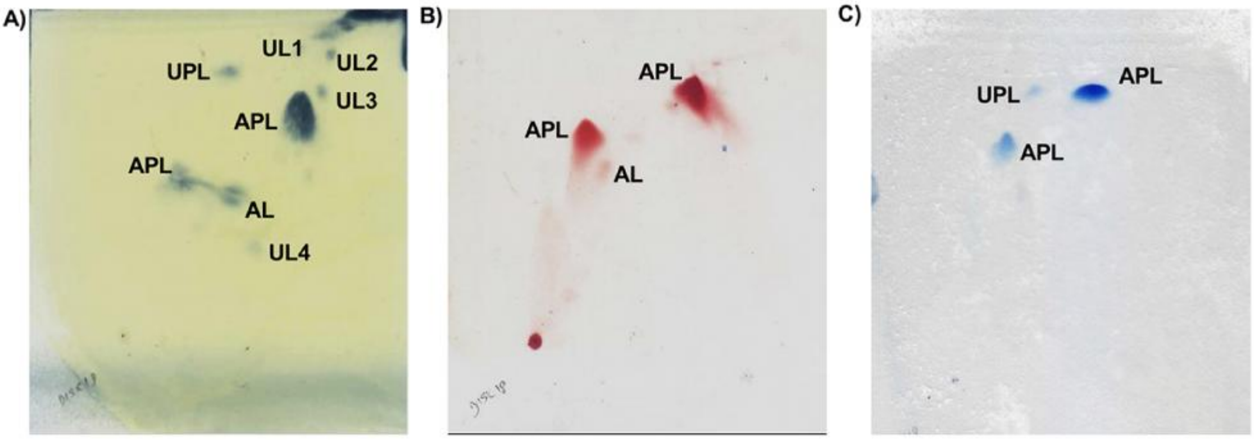

Supplementary Figure S2: Estimation of cell surface hydrophobicity of oral isolates using different solvents.

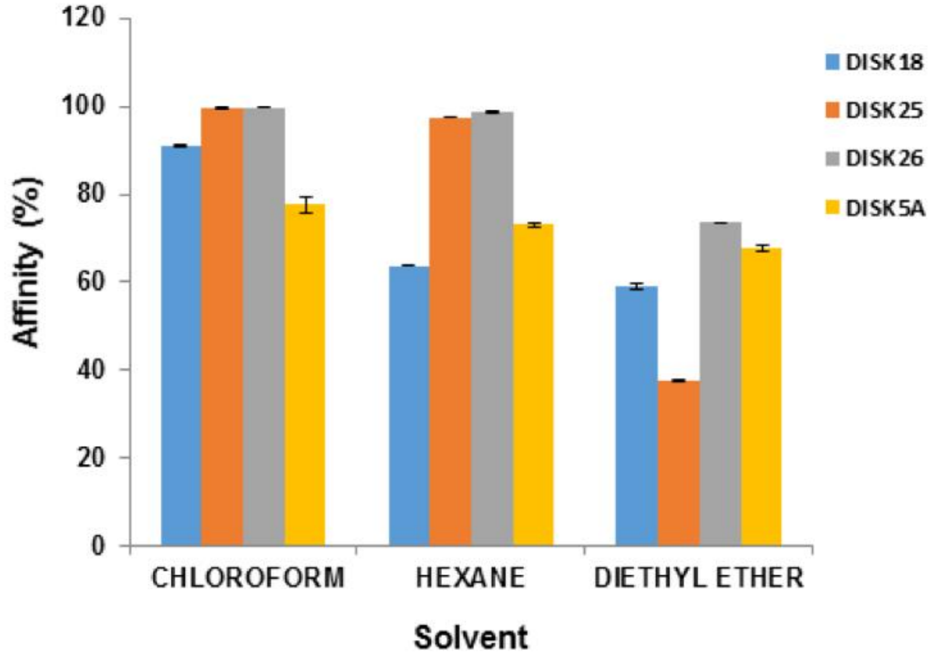

Supplementary Figure S3: Microscopic sulfide assay for VCS-producing. Microorganisms are stained black and are clearly visible under microscope as compared to control sample. (A) Cells grown in control medium. (B) Cells grown in differential medium showing black precipitation. (C) Black precipitation in differential medium upon 48 h growth.

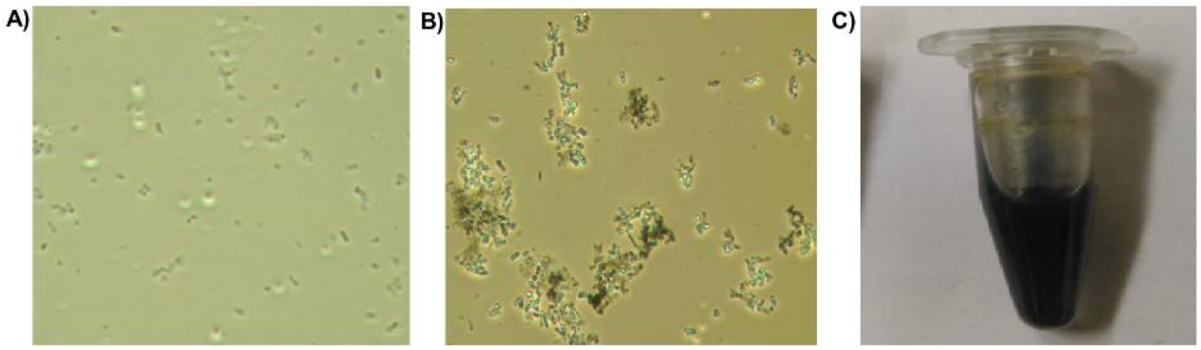

Supplement: Supplementary Information [file srep33665-s1.pdf]
